# Case report: Bruton tyrosine kinase inhibitor as therapy for chronic lymphocytic leukemia infiltrating a kidney allograft

**DOI:** 10.3389/fmed.2024.1451264

**Published:** 2024-08-29

**Authors:** Louis Stavart, Matthieu Halfon, Natacha Dewarrat, Samuel Rotman, Dela Golshayan

**Affiliations:** ^1^Transplantation Center and Transplantation Immunopathology Laboratory, Lausanne University Hospital (CHUV), Lausanne, Switzerland; ^2^Service and Central Laboratory of Hematology, Lausanne University Hospital (CHUV), Lausanne, Switzerland; ^3^Institute of Pathology, Lausanne University Hospital (CHUV), Lausanne, Switzerland; ^4^Faculty of Biology and Medicine, University of Lausanne (UNIL), Lausanne, Switzerland

**Keywords:** chronic lymphocytic leukemia, kidney transplantation, Bruton tyrosine kinase inhibitor, acute cellular rejection, case report

## Abstract

The burden of chronic lymphocytic leukemia (CLL) in the prognosis of solid organ transplant (SOT) recipients seems non-negligible. Whether transplanting a patient with previous CLL is safe or what is the optimal monitoring and treatment management after transplantation is still unclear and only based on few case series and reports. Therefore, we aimed to contribute to this understanding by reporting the first documented case of a clinically significant CLL with biopsy-proven infiltration of the kidney allograft and its successful management with a Bruton tyrosine kinase inhibitor (BTKi). We then reviewed the related literature, with a focus on CLL and kidney transplantation. Our main message is that BTKi may represent a safe and effective intervention to prevent the hazardous patient and graft outcomes of CLL in SOT patients.

## Introduction

Chronic lymphocytic leukemia (CLL) is a lymphoproliferative disorder with heterogeneous presentations, from asymptomatic to multi-organ involvements. Its incidence increases with age and familial history. Whether patients with CLL are acceptable candidates for solid organ transplantation (SOT) is still debated, with scarce available data. Patient’s and graft’s prognosis appear to differ from the clinical course of CLL, with reported poor outcomes. There are currently no recommendations on the impact and need for treatment of CLL in the context of kidney transplantation (KTx) with chronic immunosuppressive therapy. We report the first case of a clinically significant CLL with biopsy-proven infiltration of the kidney allograft and its management with a Bruton tyrosine kinase inhibitor (BTKi). We then review the literature on the occurrence and outcome of CLL in KTx recipients.

## Case report

### Clinical findings and diagnosis

We report the case of a 70-year-old woman with a 30-year history of slow-progressing chronic kidney disease. The decline in kidney function was not fully documented (in particular, no biopsy of the native kidneys) and occurred in the context of chronic diarrhea following bowel surgery, complicated by calcium-oxalate kidney stones and possibly associated nephropathy. Her medical history was otherwise unremarkable, besides several abdominal surgeries including transverse colon resection for intramucosal adenocarcinoma. After 4 years of hemodialysis, she received a kidney from a 70-year-old male donor after cardio-circulatory death (DCD) following traumatic intracranial hemorrhage (last serum creatinine, sCr: 61 μmol/L). Induction therapy consisted of intraoperative administration of basiliximab followed by polyclonal rabbit antithymocyte globulin (at day 2, 3, 4, 7, 10, and 14; cumulative dose 425 mg). Maintenance immunosuppressive regimen consisted of methylprednisolone pulses followed by prednisone at tapering doses, mycophenolate mofetil (MMF), and tacrolimus (from day 17 as diuresis resumed).

**Figure 1:**
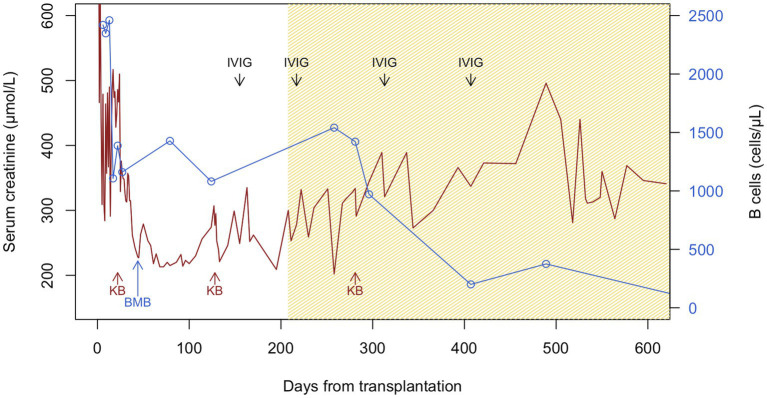
Serum creatinine and B-cell counts during follow-up after kidney transplantation. Patient suffered from delayed graft function with the last hemodialysis session on day 24, warranting the first kidney biopsy (KB, day 22), which showed acute Banff IA T-cell-mediated rejection and interstitial infiltrates of monoclonal B cells. After intravenous pulses of methylprednisolone, serum creatinine (sCr) improved until day 61. However, sCr plateaued and then rose since day 100, warranting a second KB (day 128), which showed possible borderline rejection and persistent monoclonal B-cell clusters. Patient was again treated with intravenous methylprednisolone pulses. Together with the peripheral blood immunophenotyping and B-cell counts, the bone marrow biopsy at day 44 and the characterization of the interstitial monoclonal B-cell infiltrates in the first KB, the diagnosis of chronic lymphocytic leukemia was retained. Given the increase in sCr, BTKi was introduced since day 208. The transient rise in B-cell counts post-BTKi is called *redistribution lymphocytosis* resulting from inhibition of B-cell adhesion molecules and signaling chemokines. Despite plummeting of peripheral B-cell counts, there was no significant improvement in graft function. The third KB (day 281) showed progression of interstitial fibrosis, but no signs of rejection and almost no infiltrating monoclonal B cells. Polyclonal immunoglobulins were given at replacement doses (at day 155, 217, 313, and 407 post-transplantation) for hypogammaglobulinemia. Brown and blue lines represent sCr and B-cell counts, respectively. The hatched brown section of the graph represents time under BTKi treatment. BMB, bone marrow biopsy; BTKi, Bruton tyrosine kinase inhibitor; IVIG, intravenous immunoglobulins; KB, kidney transplant biopsy.

**Figure 2:**
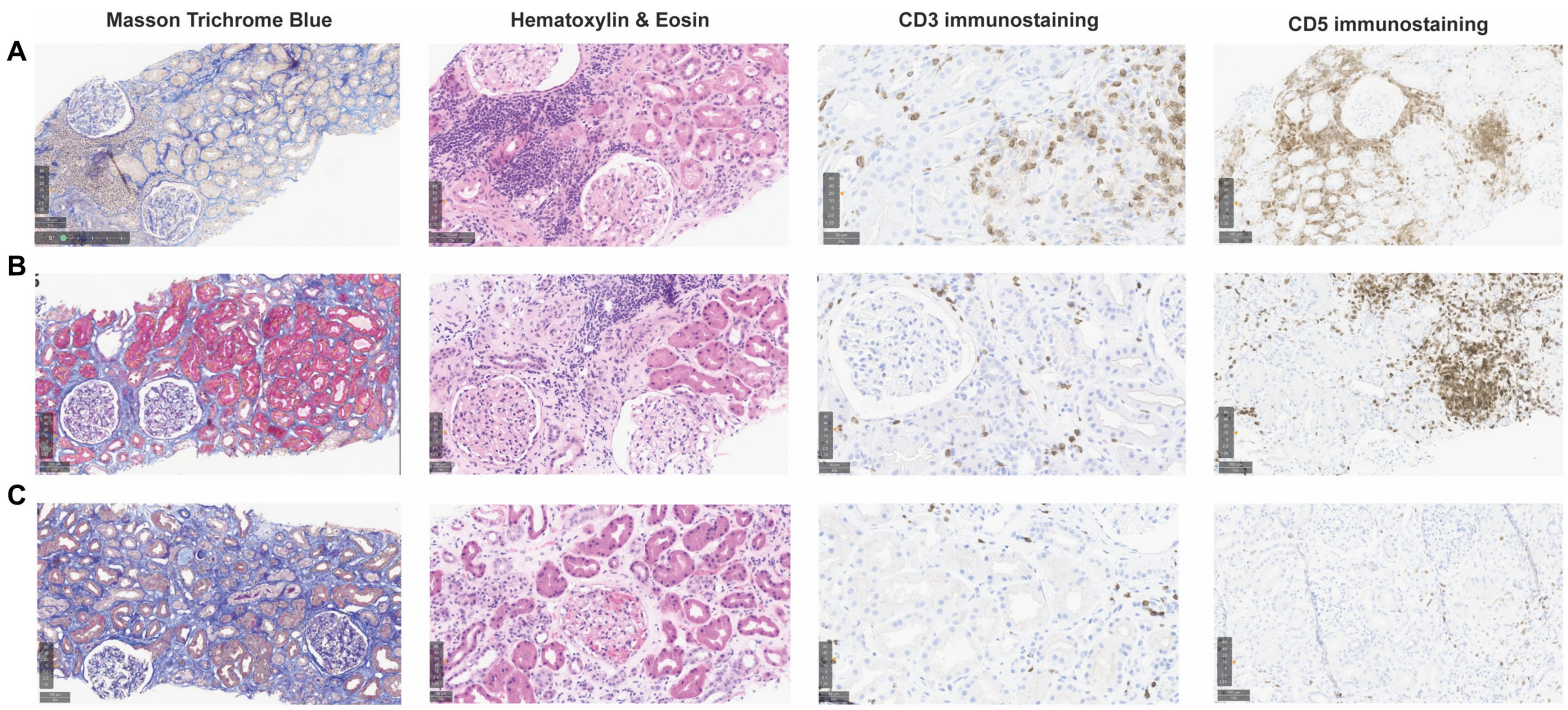
Consecutive kidney allograft biopsies during follow-up after kidney transplantation. Graft biopsies were performed at different time points during follow-up and are shown after staining with Masson Trichrome Blue (MTB, 10x), Hematoxylin & Eosin (HE, 15x), and CD3 and CD5 immunohistochemistry (IHC) staining (20x and 10x, respectively). **(A)** First biopsy (day 22) showing intact glomeruli and 10–20% interstitial fibrosis (MTB), CD3+ T-cell infiltrates associated with tubulitis corresponding to a Banff IA acute T-cell-mediated rejection (HE, CD3 IHC), and interstitial clusters of CD5+ monoclonal B cells (HE, CD5 IHC). **(B)** Second biopsy (day 128, 3 months later), showing intact glomeruli and relatively stable 10–20% interstitial fibrosis (MTB), persistant mononuclear CD5+ cells clusters (HE, CD5 IHC) and small CD3+ clusters compatible with borderline rejection (HE, CD3 IHC). **(C)** Third biopsy (day 281, 5 months later) showing atrophic tubules and increased interstitial fibrosis 20–30% (MTB), almost no monoclonal B cells (HE, CD5 IHC) and no signs of T-cell-mediated rejection (HE, CD3 IHC).

Initial follow-up was characterized by prolonged delayed graft function (DGF) with a last hemodialysis session on postoperative day 24. sCr levels then plateaued for 10 days around 350 μmol/L without proteinuria, and progressively decreased to 218 μmol/L on day 61 (estimated glomerular filtration rate, eGFR, 19 mL/min/1.73 m^2^) (Figure 1). During this early in-hospital follow-up, the patient presented two episodes of urinary tract infection treated with antibiotics: a graft pyelonephritis (day 18), and a cystitis (day 41). Due to the slow recovery of graft function, repeated renal imaging was performed using Doppler-ultrasound and scintigraphy, showing no surgical complications but signs of acute tubular necrosis. No donor-specific anti-human leukocyte antigen antibodies (DSA) were detected. A first allograft biopsy (day 22) revealed signs of Banff IA acute T-cell-mediated rejection (TCMR, Banff adequate-t1-i1-ti1-ptc0-v0-cv0-g0-cg0-mm1-ci1-ct1-ah0-aah0-c4d0-pv0-iIFTA1), with no criteria for acute antibody-mediated rejection (Figure 2A). We therefore administered pulse intravenous methylprednisolone (500 mg/pulse, total 6 doses).

Interestingly, besides TCMR, interstitial CD5+ monoclonal B-cell clusters were found in this first post-KTx biopsy. In fact, the patient was known to have chronic elevation in leukocytes counts at around 25–30 G/L (20 G/L on the day of KTx before the administration of immunosuppressive treatments) without specific investigations (in particular, no bone marrow biopsy) and a precise diagnosis, for many years before KTx. Clinical examination did not reveal any lymphadenopathy or organomegaly. Laboratory investigations showed severe hypogammaglobulinemia (IgG 2.10 g/L) and a predominance of B lymphocytes in the peripheral blood (2,500 cells/mm^3^, representing 80% of all lymphocytes), with >10% disrupted smudge cells (Gumprecht shadow) on peripheral smear. Detailed peripheral blood phenotyping showed that B lymphocytes expressed a CLL-like antigen pattern (CD5+, CD11c+, CD19+, CD20+/low, CD23+, CD43+, CD79b+, surface IgM+/low, CD200+; CD10−, CD103−, CD38−, CD123−). The bone marrow biopsy (day 44) disclosed: (a) interstitial monoclonal B lymphocytosis around 10% with a CLL-like immunophenotype: PAX5+, CD5+, CD19+, CD20+; chromosome 12 trisomy; absence of TP53 mutation; unmutated immunoglobulin heavy chain variable gene, IGHV (IGHV 3–30/ IGHD 3–3*01/ IGHJ 5*02, 0% somatic hypermutation), (b) mild pancytopenia, (c) mild polytypic plasmacytosis. Hepatitis B and C viruses, human immunodeficiency virus, and cytomegalovirus serologies were negative at KTx. The positron emission tomography-computed tomography scanner was unremarkable. Beta-2-microglobulin level was at 6.58 mg/L. Thus, the diagnosis of Binet stage A (Rai stage 0) CLL was made, according to the latest international guidelines (1) with CLL-International Prognostic Index calculated at 5 points (high-risk category, 63.3% 5-year survival) (2).

Clinically, sCr increased progressively from day 100 onwards. Radiological findings, polyomavirus BK viremia, and DSA remained negative. A second kidney biopsy (day 128) revealed: (a) borderline TCMR, (b) moderate arteriolosclerosis, and (c) focal interstitial infiltration (10–20% total surface) by CLL-like B lymphocytes (PAX5+, CD5+, CD20+, CD23+, surface IgM-; cyclin D1 negative) (Figure 2B). The interstitial infiltration had an XX profile while kidney epithelial cells were XY by interphase fluorescence *in situ* hybridization, showing that CLL infiltration was not donor-derived but originated from the patient. We again administered 4 pulses of intravenous methylprednisolone.

### Therapeutic intervention and outcome

As prolonged DGF, the plateau phase and novel increase of sCr after treatment of TCMR may have been explained by CLL graft infiltration, we introduced a BTKi (oral ibrutinib 140 mg once every other day) since day 208 (3, 4). Immunosuppression was modified with discontinuation of MMF and lower tacrolimus trough levels. To prevent opportunistic infections (5, 6), our patient received prophylaxis with sulfamethoxazole-trimethoprim, valacyclovir, and posaconazole, together with regular intravenous immunoglobulin replacement (0.5 mg/kg, at day 155, 217, 313, and 407). As sCr stabilized but did not decrease significantly, a third biopsy (day 281) was performed and revealed: (a) moderate interstitial fibrosis (20–30%), (b) no signs of rejection, and (c) absence of CLL-like B lymphocytes infiltration (PAX5-, CD5-, CD23-) (Figure 2C).

Approximately 1 year after BTKi introduction (18 months after KTx), sCr stabilized at around 280–300 μmol/L with no significant albuminuria. During follow-up, the patient did not experience complications related to BTKi treatment. The evolution of B-cell counts and sCr after KTx is displayed in Figure 1. Unfortunately, the patient had several episodes of acute kidney injury (AKI) following severe non-infectious watery diarrhea (context of hemicolectomy), but renal function mostly returned to baseline values after rapid volume management.

## Discussion

CLL is a lymphoproliferative disorder, characterized by the accumulation of monoclonal mature B lymphocytes (7). It prevails in adults from western countries, especially among men and elderly patients >65 years of age (6). Following the latest International Workshop on CLL guidelines, CLL is defined by a 3-month sustained elevation in the peripheral blood of clonal B lymphocytes >5,000 cells/mm^3^ with typical morphological and immunophenotypic features, or by the presence of cytopenias related to clonal B-cell infiltration of the bone marrow (even with peripheral count <5,000 cells/mm^3^). In other cases, if clonal B-cell counts is <5,000 cells/mm^3^, lymphadenopathy establishes the diagnosis of small lymphocytic lymphoma (SLL); and the absence of lymphadenopathy, clinical organomegaly or related symptoms establishes the diagnosis of monoclonal B lymphocytosis (MBL). In our patient, CLL diagnosis was retained based on the documented bone marrow clonal B-cell infiltration, peripheral blood immunophenotypic analysis showing lymphocytosis with clonal CLL-like B-cell expansion regardless of the peripheral B-cell counts (maximum 2,500 cell/mm^3^ in our case) in a patient under immunosuppressive therapy, and symptomatic organ involvement (i.e., graft infiltration) (1, 3).

Despite advances in understanding the pathogenesis of CLL (7), its clinical course remains unpredictable, ranging from indolent in 30% of cases to severe multi-organ involvement. Two clinical staging systems (Rai and Binet) are commonly used to stratify CLL according to lymph nodes, spleen, and liver enlargement, as well as peripheral cytopenias (8, 9). A more recent score, the International Prognostic Index for CLL (IPI-CLL), implementing age, beta-2-microglobulin levels, and specific gene mutations status has improved prognosis estimation (2). Our patient was classified as low-risk by clinical scores (Binet A and Rai 0) but as high-risk by the IPI-CLL score.

Native kidney involvement of CLL seems common and mostly without severe consequences, but the underlying mechanisms of this tropism towards the kidney are unclear. Barcos et al. performed 109 autopsies on CLL patients and found that kidneys were the most frequently infiltrated organs (63% of cases) after liver, spleen, and lymph nodes (10). Other autopsy studies reported asymptomatic kidney involvement in up to 90% of cases. The infiltration was described as mild to moderate, in defined foci, involving the cortex and the corticomedullary junction, with increased fibrosis and patterns of chronic interstitial nephritis (11, 12). Nephrotic syndrome and AKI were the most prevalent biopsy indications in a case series of CLL patients and in the 44-case retrospective study of the Mayo Clinic (13, 14). The latter found that >70% of the pathological findings could be directly related to CLL, with the most frequent lesions being membranoproliferative glomerulonephritis, followed by CLL infiltration as primary etiology, minimal change disease, or acute interstitial nephritis. The six patients with CLL infiltration as primary etiology had moderate to severe kidney failure at the time of biopsy (sCr range 149–750 μmol/L), and < 5,000 peripheral blood leucocytes/mm^3^. Renal impairment did not appear to be associated with the severity of CLL infiltration, and sCr improved in all patients who achieved a response to treatment (i.e., rituximab, cyclophosphamide, vincristine, and/or prednisone) (14). The largest case series (15 CLL patients in France) displayed a similar distribution of renal biopsy indications and pathological findings, and also observed an improvement in renal function if CLL could be successfully treated (15). Our patient was listed for KTx with no diagnostic biopsy of her native kidneys while she probably already had CLL with possible tropism to the kidneys.

The potential impact of CLL graft infiltration is even more equivocal. The first case of KTx in a patient with previously diagnosed CLL was reported by Davin et al. (16). CLL did not appear to significantly impact graft function (stable sCr after KTx) but the patient required multiple hospitalizations for bacterial and viral infections during the 2-year follow-up. Authors did not mention any biopsy or allograft infiltration (16). Thirty years later, d’Ythurbide et al. drew attention on potential disastrous outcomes, by describing the disease course of four deceased-donor KTx recipients for whom the diagnosis of Binet stage A CLL was made either before or after transplantation. During the 4-year follow-up, CLL progression was found in three patients: the first exhibited reduced eGFR with massive CLL infiltration on biopsy and a growing number of lymphadenopathies, despite chlorambucil treatment; the second lost his graft 14 months after KTx also with massive CLL infiltration on biopsy despite low-dose irradiation of the graft, rituximab, and chemotherapy; and the last patient developed thrombopenia, splenomegaly and thoracic lymphadenopathies, with a stable sCr, but died 4 years after transplantation from sepsis. One patient developed Kaposi sarcoma and B-cell lymphoma, and all four patients experienced severe infectious complications. The higher susceptibility to infections was explained by the combination of post-KTx immunosuppressive regimen and inherent immune defects (cytopenia, hypogammaglobulinemia) related to CLL (17). At an extended follow-up (total 6.5 years), two other patients had died from CLL progression, and the last living patient had lost her graft 4.5 years after KTx with diffuse CLL infiltration and severe interstitial fibrosis on biopsy. The authors reasserted the hazardous prognosis of these patients along with a high rate of hospitalizations (18). Strati et al. reported their experience at the Mayo Clinic with two living-donor KTx recipients with Rai stage 0 CLL. The first patient was admitted four times for severe infections, lost his graft 5 years after KTx and died 1.5 years later while back on dialysis. Graft biopsy showed recurrence of the underlying glomerulonephritis and a minimal incidental CLL infiltration. The second had a good outcome, without CLL progression or graft dysfunction needing a biopsy (19). In their large cohort of immunotactoid glomerulopathy (ITG), Nasr et al. briefly mentioned one patient who had CLL and membranous-pattern recurrent ITG after KTx, but who entered in complete remission after chemotherapy (i.e., fludarabine, cyclophosphamide and rituximab) (20).

Dierickx et al. detailed a case of CLL-like B-lymphocytes graft infiltration in a KTx recipient without cytopenia or lymphocytosis, who had a relatively good outcome without any therapy (21). However, the clinical features seemed more consistent with the diagnosis of SLL (1). Similarly, Romero et al. described event-free 3-year follow-up of SLL in a patient after simultaneous kidney-pancreas transplantation (22). These cases highlight that SLL may have limited clinical consequences after transplantation compared to CLL. Similarly, there was neither transformation to CLL or lymphoma, nor graft failure among the four reported SOT patients with MBL phenotype during follow-up from 0.8 to 11.5 years (19, 23, 24). Many advocated that MBL had little impact on kidney function or malignancy outcomes in KTx patients, but that close monitoring should be performed as in non-transplanted patients (19, 23). Based on those cases, the American Society of Transplantation released a consensus statement in 2021 recommending no waiting time for SOT candidates with MBL and 2 to 3-year interval after CLL treatment (25). Finally, no clinically relevant transmission of CLL was observed in SOT recipients of the only two reported cases of a donor with CLL-like disease, which was SLL in both cases (26, 27). Accordingly, sex chromosome profiling on our patient’s allograft biopsy excluded this possibility.

Besides renal interstitial infiltration by lymphomatous cells (interstitial nephritis), CLL has been associated or directly implicated in glomerular diseases with various patterns of glomerular injury, mainly, in order of frequency, membranoproliferative glomerulonephritis, minimal change disease, membranous glomerulopathy, and ITG (14). CLL could also potentially drive the risk of incident glomerulonephritis or recurrence in the allograft (17, 19) and in native kidneys (28). In our case, there was no glomerular lesions on the graft biopsies (light microscopy) and no deposits (by immunofluorescence and electron microscopy). Early graft interstitial infiltration by monoclonal B cells, as witnessed by day 22-biopsy, appeared to be concomitant to DGF and acute TCMR. KTx is inevitably associated with some degree of AKI as a consequence of ischemia–reperfusion injury, which is more severe after DCD KTx inducing significant intragraft inflammation. Local upregulation of cytokines and chemokines triggers the recruitment and activation of innate and adaptive immune cells. Thus, in our patient’s case, severe ischemia–reperfusion injury could have promoted intragraft recruitment of immune effector cells and acute rejection. Besides the onset of TCMR, it may also have facilitated early graft infiltration by monoclonal B cells (6). It was indeed shown that high serum levels of CXCL13, a B-cell-attracting chemokine released by T cells, correlated with B-cell interstitial clusters in renal biopsies of treatment-resistant TCMR (29). The crosstalk between T and B cells within the allograft would have further exacerbated harmful immune activation loops, leading to chronic interstitial inflammation and fibrosis, and chronic kidney dysfunction (30). Still, there was no allusion to rejection in d’Ythurbide et al. case series detailed above, while two patients of Strati et al. experienced acute rejection (at 1 and 61 months after KTx, respectively) (17, 19).

According to the European CLL management guidelines, our patient was initially monitored without therapy (watch-and-wait strategy) until the documentation of an active disease (3). sCr elevation concomitant to clonal B-cell infiltration on allograft biopsies was considered as a plausible functional extra-nodal involvement of CLL, warranting front-line therapy (3). Following knowledge at the time for native kidneys, ibrutinib was chosen on the basis of an unmutated IGHV and absence of TP53 mutation in a relative fit older patient (3). Ibrutinib selectively inhibits a non-receptor tyrosine kinase present on B lymphocytes, therefore blocking their survival, proliferation, and migration (5). According to previous data under alkylating agents, high-risk CLL patients had an estimated median life expectancy <2 years, whereas survival at 7 years under BTKi is currently reported to be >75% (6, 31). Based on improved outcomes showed by randomized control trials, treatment of CLL patients has indeed shifted during the last decade from chemotherapy-based regimens (together with anti-CD20 antibodies) to targeted agents only, like BTKi (5, 6). Interestingly, we observed a transient rise of B cells in peripheral blood following BTKi introduction, compatible with “redistribution lymphocytosis,” as previously described in the early phase of treatment (Figure 1) (5, 6). There is currently no recommendation regarding the assessment of the minimal residual disease status or the duration of therapy. We however decided to maintain treatment until B cells felt within normal range or sCr returned to baseline. According to current data, during the maximum 8-year follow-up (median 6.9 years) of CLL patients treated by ibrutinib, the medication was deemed safe and well tolerated (31). Besides infections, ibrutinib increased the risk for hypertension (particularly in association with chronic kidney disease), atrial fibrillation, and low-grade bleeding (6, 32). Seventy-three days after the introduction of BTKi therapy, our patient’s third post-KTx biopsy revealed no signs of CLL infiltration.

Finally, although we documented significant improvement of CLL infiltration in a kidney allograft following BTKi, the real burden of CLL in our case’s graft function remains uncertain. First, characteristics related to the donor organ might have contributed to the severity of initial DGF, independent of CLL infiltration. DGF is more common in DCD KTx and is associated with greater risks of acute rejection and worse baseline eGFR (33). Second, due to the lack of a native kidney biopsy, we do not know if the initial nephropathy was also linked to CLL infiltration. Nevertheless, allograft CLL involvement was observed early after KTx (i.e., by day 22), possibly triggered by ischemia–reperfusion injury and subsequent local immune response, or purely by the known high propensity of CLL monoclonal B cells to infiltrate the kidney. Then, the real impact of CLL on progressive graft dysfunction in the follow-up stays unanswered (14). Early graft biopsies indeed showed alternative diagnoses such as TCMR. If KTx patients with CLL are at higher risk for TCMR, or if TCMR drives the risk of CLL infiltration in the allograft, warrants further studies. At later stages, sCr did not improve significantly despite the disappearance of monoclonal B-cell clusters on the last biopsy and reduced B-cell counts in peripheral blood. This could be partly explained by progressive interstitial fibrosis despite targeted BTKi therapy, triggered by initial CLL infiltration and possible episodes of acute tubular necrosis in the context of chronic diarrhea. Thus, we cannot ascertain treatment benefits in our case and no specific recommendations exist. We found only eight reported cases of KTx recipients with CLL of whom only four with documented allograft infiltration. Almost every patient displayed poor outcomes, even with chemotherapy and rituximab, and the implication of CLL or its treatment in those outcomes remained uncertain.

## Conclusion

In summary, we report the first case of CLL infiltration in a kidney allograft successfully controlled by a BTKi. We followed current guidelines recommending first-line therapy when active disease is diagnosed (e.g., functional extra-nodal involvement). As emphasized by Strati et al., therapeutic management was based on renal biopsies, and discussed by a multidisciplinary team of transplant nephrologists and hematologists (14). Overall, KTx patients with CLL are at increased risk of adverse outcomes regarding (a) the lymphoproliferative disorder: CLL progression, tissue infiltration, and transformation; (b) the patient: infections, hospitalizations, and death; and (c) the allograft: AKI, glomerulonephritis, and graft failure. Counterintuitively, the prognosis of those patients and their allograft seems to differ from the CLL clinical course. To date, data on the impact and treatment of CLL in KTx are scarce. Nevertheless, regarding their good tolerance and relative favorable safety profile providing proper anti-infectious prophylaxis, treatment by BTKi may be an acceptable alternative to prevent the hazardous patient and graft outcomes reported in the literature.

## Patient perspective

The patient felt unsettled that her hematologic condition, dismissed as benign before KTx, could play a significant role in graft outcome. She appreciated the multidisciplinary management aiming at preserving the function of her allograft. Oral administration and absence of side effects of ibrutinib contributed to her compliance and wellbeing.

## Data Availability

The original contributions presented in the study are included in the article/supplementary material, further inquiries can be directed to the corresponding author.
